# Exploring methods the for selection and integration of stakeholder views in the development of core outcome sets: a case study in reconstructive breast surgery

**DOI:** 10.1186/s13063-016-1591-y

**Published:** 2016-09-23

**Authors:** Shelley Potter, Sara T. Brookes, Christopher Holcombe, Joseph A. Ward, Jane M. Blazeby

**Affiliations:** 1 Bristol Centre for Surgical Research, School of Social and Community Medicine, University of Bristol, 39 Whatley Road, Clifton, Bristol, UK; 2 Linda McCartney Breast Care Centre, Royal Liverpool and Broadgreen University Hospitals NHS Trust, Liverpool, UK; 3 Division of Surgery, Head and Neck, University Hospitals Bristol NHS Foundation Trust, Upper Maudlin Street, Bristol, UK

**Keywords:** Core outcome sets, Stakeholder selection, Delphi, Breast reconstruction, Methodology

## Abstract

**Background:**

The development and use of core outcome sets (COSs) in trials may improve data synthesis and reduce outcome reporting bias. The selection of outcomes in COSs is informed by views of key stakeholders, yet little is known about the role and influence of different stakeholders’ views during COS development. We report an exploratory case study examining how stakeholder selection and incorporation of stakeholders’ views may influence the selection of outcomes for a COS in reconstructive breast surgery (RBS). We also make recommendations for future considerations.

**Methods:**

Key stakeholder groups and subgroups were identified from the literature and expert opinion by the COS management group. They included health care professionals, subdivided by profession (breast and plastic surgeons, specialist nurses and psychologists) and patients, subdivided according to type of surgery received, timing of reconstruction, time since surgery and patient age. All participated in a survey in which they were asked to prioritise outcomes. Outcomes were prioritised using a 9-point scale from 1 (not important) to 9 (extremely important). The proportion of (1) all participants, ignoring stakeholder group (single heterogeneous panel analysis), (2) ‘professional’ and ‘patient’ groups separately (two heterogeneous panels), ignoring prespecified subgroups and (3) each participant subgroup separately (multiple homogeneous panel analysis) rating each item ‘extremely important’ was summarised and compared to explore how selection and integration of stakeholder views may influence outcome prioritisation.

**Results:**

There were many overlaps between items rated as most important by all groups. Specific stakeholders, however, prioritised specific concerns and a broader range of outcomes were prioritised when the subgroups were considered separately. For example, two additional outcomes were prioritised when patient and professional groups were considered separately and eight additional outcomes were identified when the views of the individual subgroups were explored. In general, patient subgroups preferentially valued additional clinical outcomes, including unplanned surgery, whereas professional subgroups prioritised additional psychosocial issues including body image.

**Conclusion:**

Stakeholder groups value different outcomes. Selection of groups, therefore, is important. Our recommendations for robust and transparent stakeholder selection and integration of stakeholder views may aid future COS developers in the design and conduct of their studies and improve the validity and value of future COS.

## Background

The careful selection of meaningful end points is essential for research to inform clinical practice and guide health policy [1, 2]. A number of systematic reviews, however, have demonstrated a lack of consistency in the way that outcomes are assessed and reported [3–8]. Heterogeneity of outcome reporting limits cross-study comparison, precludes data synthesis and introduces the possibility of reporting bias [9]. One solution to inconsistent and inappropriate outcome reporting is to develop and use core outcome sets (COSs), a scientifically agreed minimum set of outcomes to be measured and reported in all effectiveness studies of a given condition [10, 11]. Core outcome sets have been developed in a number of areas [12–15]. Their development typically involves the identification of an exhaustive list of outcomes, then prioritisation of the outcomes by stakeholders using consensus methods such as Delphi surveys [10, 16, 17]. Delphi surveys require participants to rate the importance of different outcomes in sequential questionnaires (or rounds), with responses to each outcome summarised and fed back anonymously in subsequent rounds [16]. This feedback enables participants to change their initial scores in light of others’ views. Although guidance for this process is emerging [10], the precise methodology is yet to be agreed [11, 16]. A Delphi survey may lead directly to the final COS or may inform one or more subsequent consensus meetings at which the final core set is agreed [10].

Stakeholders are critical to COS development since it is their views which inform the final core set. If important stakeholders are not included, key outcomes may be omitted [18] meaning that the COS is of little future value. Recommendations for Delphi surveys suggest that involvement of health professionals and patients is essential for developing a COS for pragmatic trials [10, 16]. Yet, within health professional and patient groups there are likely to be important subgroups whose views should be considered. For example, health professionals might include surgeons, physicians, nurses, physiotherapists and psychologists, each of whom may have different views. There are currently few recommendations as to how ‘key’ stakeholder groups and subgroups should be defined and selected [14].

There is also debate as to how stakeholders’ views should be amalgamated during the Delphi process [18]. One approach is for participants to be considered a heterogeneous single panel, ignoring stakeholder type, when generating and presenting feedback and determining items to retain for the next stage of the consensus process. Alternatively, participants can be treated as multiple homogeneous panels with stakeholders considered to be distinct groups; feedback from each stakeholder group is generated and criteria for retaining items are based on the separate stakeholder groups [18]. Robust and transparent methods for the integration of views are vital if the value of future COSs is to be optimised, ensuring that the final COS has credibility in the relevant clinical field and among the research community.

Reconstructive breast surgery (RBS) is a complex area as there are a number of different types of reconstructive procedures that can be performed [19]. These range in complexity from expander-implant-based reconstruction to microvascular free-flap techniques. Each procedure has specific risks and benefits. For example, implant-based reconstruction is a simple procedure with a quick recovery that produces good results but may require revision over time; whereas free-flap reconstructions are longer, more complex procedures that produce excellent long-term cosmetic results, but have a longer recovery and the risk of donor-site morbidity. Patients electing to undergo different types of reconstruction may prioritise and value outcomes differently [20, 21]. Other factors, including patients’ age, whether they elect to undergo reconstruction at the time of their mastectomy or at a later date and the time elapsed since the original surgery, have also been shown to impact on which outcomes are valued [20]. Similarly, professional stakeholders in breast reconstruction include breast and plastic surgeons, clinical nurse specialists (CNS) and psychologists, and each subgroup may have different views as to what outcomes are most important.

Using RBS as a case study, the aim of this paper was to explore to what extent decisions regarding stakeholder selection and integration of stakeholder views within Delphi surveys may influence the content of a COS and to propose a framework for use in future studies. The recommendations may also inform consensus methods other than Delphi methodology.

## Methods

This research was integrated into the BRAVO study which developed a COS for RBS [15]. Full ethical approval was obtained for the study (REC-11/SW/0305).

The BRAVO study is reported in detail elsewhere [15], but in brief the study consisted of three phases: phase 1 – creation of a questionnaire with an exhaustive list of potential outcomes identified from systematic literature reviews and qualitative work with patients and health care professionals; phase 2 – two sequential surveys with 303 key stakeholders (215 patients and 88 health care professionals) using Delphi methods to prioritise outcomes; phase 3 – two consensus meetings, one with patients and one with professionals to agree the COS. This paper is informed by the initial round-1 Delphi survey conducted in phase 2.

### Stakeholder selection

Key stakeholders and important stakeholder subgroups were identified based on the literature [22–26], previous qualitative work [20, 21, 27–31] and expert opinion by the BRAVO Steering Group. Broadly, these included health care professionals and patients. The professional group was subdivided into breast surgeons, plastic surgeons, CNS and psychologists; the patient group was subdivided based on age, type and timing of reconstruction and time since surgery.

Professionals were purposively recruited from breast and plastic surgical units across the UK using the qualitative approach of maximum variation sampling [32]. Variation was sought with regard to type of centre (teaching hospital versus district general hospital), gender and duration of practice to ensure a comprehensive representation of views. These variables were not considered to constitute specific professional subgroups in this study as there is no evidence to suggest that these factors would influence item prioritisation in a Delphi process. A priori, the aim was to recruit 30 breast surgeons, 30 plastic surgeons, 30 CNS and 10 psychologists. This ratio was chosen because surgeons and specialist nurses are involved in the decision-making process for all patients whereas psychologists have an important role but do not see every patient prior to surgery.

Patients were purposively sampled from three centres (Bristol, Liverpool and Glasgow). Qualitative maximum variation sampling methodology [33] was again used to ensure that each prespecified patient subgroup was adequately sampled. These groups included women undergoing each of the four main types of RBS (expander/implant, latissimus dorsi flap, abdominal flap reconstruction and therapeutic mammaplasty); those who had undergone reconstruction at the same time as their mastectomy (immediate reconstruction) or as a delayed procedure; women who had had surgery in the recent past, defined as occurring within 24 months of questionnaire completion, and those who had had surgery more than 2 years prior to participating in the study; and young (under 45 years), middle-aged (45–60 years) and older women (over 60 years). The subgroups were selected based on the findings of earlier qualitative work [20, 21, 28] which suggested that each factor may influence outcome prioritisation. This approach was chosen to ensure that the widest breadth of views was included in the COS development process. Based on this sampling strategy, it was anticipated that approximately 200 patients would be recruited to the study.

#### Questionnaire survey

Questionnaires asked participants to score the importance of each of 34 outcomes on the 1–9 point scale (1 ‘not important, 9 ‘extremely important’) proposed by the GRADE Group (www.gradeworkinggroup.org) and recommended by the COMET Initiative [10]. Outcomes considered short- and long-term complications, symptoms following surgery, psychosocial issues, practical issues and cosmesis. Nonresponders were sent a reminder 3 weeks later. Batches of invitations were sent until the desired sample size was achieved or until the sample pool had been exhausted.

### Data analysis

The number and percentage of participants rating an item ‘extremely important’ (score of 9) were calculated for each item; items were then ranked and the ‘top 10’ identified. This was done in three different ways, for: (1) the whole group, ignoring stakeholder status (single heterogeneous panel analysis), (2) broad ‘patient’ and ‘professional’ groups, ignoring stakeholder subgroups (two heterogeneous panels) and (3) prespecified professional (breast surgeons, plastic surgeons, CNS and psychologists) and patient (age, type and timing of surgery and time elapsed since surgery) stakeholder subgroups separately (multiple homogeneous panels). The top 10 items for the whole group, each broad stakeholder group and each stakeholder subgroup were compared to explore how stakeholder selection and integration of stakeholder views may influence the contents of a COS. Stata version 14 was used for all analyses [34].

## Results

One hundred and fifty-six professionals were invited to participate of whom 88 (56.4 %) completed and returned the questionnaire. This included 40 breast surgeons, 21 plastic surgeons, 20 CNS and seven psychologists from centres across the UK with a range of experience. Response rates were 71.4 % (40/56), 46.7 % (21/45), 44.4 % (20/45) and 63.6 % (7/11) for breast surgeons, plastic surgeons, CNS and psychologists, respectively. Fewer plastic surgeons, CNS and psychologists participated than hoped due to difficulty engaging these stakeholders; recruitment continued until the sample pool was exhausted. There was an even mix of men and women and there was a wide spread of years in post (Table 1).

**Table 1:** Demographics of participants in the BRAVO study

Professional participants	Number = 88 (%)
Gender	
Female	46 (52.3)
Profession	
Consultant breast surgeon	40 (45.5)
Consultant plastic surgeon	21 (23.9)
Clinical nurse specialist	20 (22.7)
Psychologist	7 (8.0)
Time in post	
<5 years	18 (20.5)
5–10 years	30 (34.1)
10–20 years	29 (33.0)
>20 years	8 (9.1)
Missing	3 (3.4)
Patient participants	(Number = 215) (%)
Centre	
Bristol	77 (35.8)
Liverpool	74 (34.4)
Glasgow	64 (29.8)
Age^a^	
<45 years	21 (9.8)
5–65 years	166 (77.2)
>65 years	28 (13.0)
Median age (range)	54 (29–76)
Time since breast reconstruction^b^	
0–24 months	72 (33.5)
25–48 months	88 (40.9)
>48 months	49 (22.8)
Missing	6 (2.8)
Median (range, months)	33 (4–97)
Timing of surgery	
Immediate reconstruction	110 (51.2)
Delayed reconstruction	80 (37.2)
Therapeutic mammoplasty	25 (11.6)
Type of surgery	
Implant-based reconstruction	54 (25.1)
Latissimus dorsi flap	59 (27.4)
Abdominal flap	74 (34.4)
Therapeutic mammoplasty	25 (11.6)
Other^c^	3 (1.4)
Education	
Compulsory only	65 (30.2)
Additional education	139 (64.7)
Missing	11 (5.1)
Marital status	
Single	23 (10.7)
Married/living with partner	153 (71.2)
Separated or divorced	28 (13.0)
Widowed	6 (2.8)
Missing	5 (2.3)
Employment status	
Full- or part-time employment	130 (60.5)
Homemaker/housewife	17 (7.9)
Retired	45 (20.9)
Not working	16 (7.4)
Missing	7 (3.3)

^a^At the time of breast reconstruction; ^b^At the time of entering the study; ^c^Patients undergoing bilateral complex surgery who could not be classified into any one group

Four hundred and thirty-four patients from three centres were invited to take part in the study of whom 242 (55.8 %) consented to participate and 215 (49.5 %) completed and returned the questionnaire. A good representation of each of the prespecified subgroups was obtained with the exception of women undergoing therapeutic mammaplasty (Table 1). Despite recruiting until the sample pool was exhausted, this group remained relatively under-represented with only 11.6 % (*n* = 25) of respondents undergoing this procedure type.

Table 2 presents all 34 outcomes included in the questionnaire and indicates the top 10 outcomes prioritised as ‘extremely important’ (score of 9) by: (1) the whole group, ignoring stakeholder status, (2) broad ‘patient’ and ‘professional’ groups ignoring stakeholder subgroups and (3) professional and patient stakeholder subgroups separately. Differences in the items prioritised are summarised in Table 3.

**Table 2:** Top 10 concerns prioritised by stakeholder groups in the BRAVO study

Items prioritised in ‘top 10’ concerns	All stakeholders combined	All HCPs	Professional subgroup				All patients	Patient subgroups										
								Type of reconstructive surgery				Timing of reconstruction		Age of women undergoing BR (years)			Time since BR	
			Breast surgeon	Plastic surgeon	CNS	Psych		Implant-based	LD flaps	Abdo flap	TM	IBR	DBR	<45	45–60	>60	<24 months post-op	>24 months post-op
Short-term complications																		
Systemic complications																✓	✓	
Bleeding-related complications									✓			✓		✓			✓	✓
Wound-related complications			✓				✓	✓	✓	✓	✓	✓		✓	✓	✓	✓	✓
Implant-related complications	✓	✓	✓	✓		✓	✓	✓	✓	✓	✓	✓	✓	✓	✓	✓	✓	✓
Flap-related complications	✓	✓	✓	✓	✓	✓	✓		✓	✓	✓	✓	✓	✓	✓	✓	✓	
Major complications	✓	✓	✓	✓	✓	✓	✓	✓	✓	✓	✓	✓	✓	✓	✓	✓	✓	✓
Long-term complications																		
Long-term implant-related complications				✓				✓				✓		✓		✓		
Long-term flap-related complications																		
Donor-site complications																✓		
Unplanned surgery for any reason				✓			✓		✓	✓	✓	✓	✓	✓	✓	✓	✓	
Symptoms following reconstructive breast surgery																		
Fatigue																		
Breast symptoms																		
Arm and shoulder symptoms																		
Implant-related symptoms																		
Donor-site symptoms																		
Psychosocial issues																		
Self-esteem	✓	✓	✓	✓	✓		✓	✓			✓		✓		✓			
Body image	✓	✓	✓	✓	✓	✓		✓					✓					✓
Normality	✓	✓	✓	✓	✓	✓	✓	✓	✓	✓			✓	✓	✓			✓
Emotional well-being	✓	✓		✓	✓	✓	✓	✓			✓		✓					
Sexual well-being				✓	✓	✓												
Quality of life	✓	✓	✓	✓	✓	✓	✓	✓	✓	✓	✓	✓	✓	✓	✓		✓	✓
Practical issues																		
Physical well-being																		
Recovery time																		
Duration of the procedure																		
Time to completion of breast reconstruction																		
Number of procedures required																		
Clothing issues																		
Financial issues																		
Economic issues																		
Cosmesis																		
Patient-reported cosmetic outcome	✓	✓	✓		✓	✓	✓	✓	✓	✓	✓	✓	✓	✓	✓	✓	✓	✓
Objective cosmetic outcome										✓				✓				
Cosmetic outcome assessed by patient’s partner																		
Women’s cosmetic satisfaction	✓	✓	✓		✓	✓	✓	✓	✓	✓	✓	✓	✓	✓	✓	✓	✓	✓

*Abdo flaps* abdominal flap reconstruction, *BR* breast reconstruction, *CNS* clinical nurse specialists, *DBR* delayed breast reconstruction, *HCP* health care professionals, *IBR* immediate breast reconstruction, *LD* latissimus dorsi flaps, *post-op* time post-operatively, *psych* psychologists, *RBS* reconstructive breast surgery, *TM* therapeutic mammaplasty

**Table 3:** Summary of outcomes prioritised by using different approaches to the integration of stakeholders’ views

Single heterogeneous panel analysis (outcomes prioritised by all participants combined ignoring stakeholder groups) (*n* = 10)	Two heterogeneous panel analysis (outcomes prioritised considering professionals and patients separately but ignoring prespecified subgroups) (*n* = 12)	Multiple homogeneous panel analysis (outcomes prioritised considering professional and patients subgroups separately) (*n* = 18)
Short-term complications (*n* = 3)	Short-term complications (*n* = 4)	Short-term complications (*n* = 6)
Implant-related complications Flap-related complications Major complications	*Wound-related complications (Pt)* Implant-related complications (Both) Flap-related complications (Both) Major complications (Both)	*Systemic complications (Pt-SG3, Pt-SG4*) *Bleeding-related complications (Pt-SG1, Pt-SG2, Pt-SG3, Pt-SG4*) *Wound-related complications (P-SG) (Pt-SG1, Pt-SG2, Pt-SG3, Pt-SG4*) Implant-related complications (all) Flap-related complications (all) Major complications (all)
Long-term complications (*n* = 0)	Long-term complications (*n* = 1)	Long-term complications (*n* = 3)
	*Unplanned surgery for any reason (Pt)*	*Long-term implant-related complications (P-SG) (Pt-SG1, Pt-SG2, Pt-SG3*) *Donor-site complications (Pt-SG3*) *Unplanned surgery for any reason (P-SG) (Pt-SG1, Pt-SG2, Pt-SG3, Pt-SG4*)
Psychosocial issues (*n* = 5)	Psychosocial issues (*n* = 5)	Psychosocial issues (*n* = 6)
Self-esteem Body image Normality Emotional well-being Quality of life	Self-esteem (Both) *Body image (P)* Normality (Both) Emotional well-being (Both) Quality of life (Both)	Self-esteem (all) Body image (all) Normality (all) Emotional well-being (all) *Sexual well-being (P-SG)* Quality of life (all)
Cosmesis (*n* = 2)	Cosmesis (*n* = 2)	Cosmesis (*n* = 3)
Patient-reported cosmetic outcome Women’s cosmetic satisfaction	Patient-reported cosmetic outcome (Both) Women’s cosmetic satisfaction (Both)	Patient-reported cosmetic outcome (all) *Objective cosmetic outcome (Pt-SG1, Pt-SG3)* Women’s cosmetic satisfaction (all)

Items in italics are those identified as the top 10 priorities at each stage of the analysis by a specific stakeholder group or subgroup ; group or subgroup prioritising the outcome is given in brackets

*P* professional group, *P-SG* professional subgroup, *Pt* patient group, *Pt-SG1* patient subgroup 1 (type of reconstructive surgery), *Pt-SG2* patient subgroup 2 (timing of reconstructive surgery), *Pt-SG3* patient subgroup 3 (age of women undergoing reconstruction), *Pt-SG4* patient subgroup 4 (time since breast reconstruction)

### The whole group, ignoring stakeholder status (single heterogeneous panel)

When the participants were considered as a single heterogeneous panel ignoring any stakeholder status, the top 10 outcomes prioritised included three short-term complications, five psychosocial and two cosmetic outcomes (Table 2).

### Broad ‘professional’ and ‘patient’ groups, ignoring stakeholder subgroups (two heterogeneous panels)

#### Professionals

The top 10 outcomes prioritised by the professional stakeholder group were identical to those observed when participants were amalgamated as a whole group (see (1) in the preceding text and Table 2).

#### Patients

There were nine outcomes prioritised in the patients’ top 10 that were also prioritised by the whole group. However, two additional clinical outcomes (wound-related complications and unplanned surgery) were prioritised by the patient group (11 outcomes were included in the patients’ top 10 since there were two items rated 9 by the same percentage of patients). In addition, one outcome (body image), which was prioritised by the whole group was not prioritised by patients.

### Professional and patient stakeholder subgroups (multiple homogeneous panel)

#### Professional stakeholder subgroups

Differences were seen when the professional subgroups were considered separately compared to when all professionals were amalgamated. Four additional outcomes including one short-term complication, two long-term complications and one psychosocial issue were prioritised as top 10 concerns by at least one professional subgroup when they were considered separately. One of these items, sexual well-being, was prioritised by three of the four professional groups, but did not reach the top 10 when the four groups were combined in a broad professional stakeholder group.

Of the 34 outcomes, the four subgroups agreed on 21 items not prioritised in their top 10 (primarily symptoms after surgery and practical issues). Only five (two clinical outcomes and three psychosocial issues) were common to the top 10 items for all four professional subgroups (Table 2).

#### Patient subgroup analysis

Additional items were prioritised when patients were subdivided based on each of the predefined patient subgroups (type of procedure, timing of procedure, time since surgery and age of patient). Bleeding-related complications, which was not in the top 10 when considering all patients together, was prioritised by at least one subgroup within type of procedure, timing of procedure, time since surgery and age of patient. Long-term implant complications were prioritised by one or more subgroups within type of procedure, timing of procedure and patient age; body image was prioritised within type, timing and time since procedure; systemic complications within time since surgery and age of patient; and objective cosmetic outcome within type of procedure and age of patient (Table 2).

Within each of the patient subgroups there was a degree of disagreement. Within type of procedure, whilst all four subgroups agreed that seven out of the 34 items were in the top 10 concerns and that 19 were not, there was no consensus on the remaining eight outcomes. Within timing of procedure, both subgroups were in agreement on 27 items (seven prioritised and 20 not prioritised by both subgroups); for time since procedure, there was agreement for 30 items (seven prioritised and 23 not); and for age, all four subgroups agreed on 28 items (9 prioritised and 19 not) (Table 2).

### Recommendations for stakeholder selection and the integration of stakeholder views in the development of COSs

This work suggests that decisions regarding stakeholder selection and integration of stakeholder views within Delphi surveys may significantly influence the content of a COS. We therefore propose the following recommendations as a framework for use in future COS development studies (Table 4).

**Table 4:** Recommendations for stakeholder selection and the integration of stakeholder views in the development of core outcome sets

1	Identify all potential stakeholder groups and subgroups from the literature and expert opinion. Study Steering Group to consider all stakeholders and justify and document choice
2	The final sample size will be informed by the number of subgroups. Each subgroup should include sufficient numbers of individuals to ensure adequate representation of that group’s views. Further work is required to determine optimal numbers. Decisions regarding target numbers of individuals for each subgroup to be made, documented and justified by the study Steering Group. Any anticipated problems with recruitment and possible fall-back positions to be documented
3	Analyse prespecified stakeholder groups and subgroups separately using a multiple homogeneous panel approach. Item prioritisation criteria should then be applied to *each* individual stakeholder subgroup to determine which items are to be carried forward to the next round/consensus meeting and results presented to the Steering Group for consideration
4	Any item which meets the cut-off criteria for inclusion in *any* prespecified stakeholder subgroup should ideally be carried forward to the next round/consensus meeting irrespective of whether the item is prioritised by the group as a whole. If there are a large number of subgroups (for example, >5) or some much smaller than others, higher thresholds may be necessary for an item to be retained if prioritised by a single subgroup only or consensus may be defined with combination criteria; for example, items are only retained if inclusion criteria are met by 2 or more subgroups. Decisions regarding these criteria should be discussed and agreed by the Steering Group and the rationale for the decisions recorded
5	Any item for which uncertainty remains should be carried forward to the next Delphi round or discussed at consensus meetings. Any reason(s) why this should not be done should be discussed by the Steering Group and the justifications recorded
6	If consensus is not achieved following the Delphi process, one or more consensus meetings are advocated. Meetings should be attended by representatives of each prespecified stakeholder subgroup. Any reason(s) why this should not be done should be discussed by the Steering Group and the justifications recorded

We recommend careful selection of relevant stakeholder groups and subgroups a-priori informed by the published literature and expert opinion (Table 4). These decisions should be agreed and documented at the start of the study and reported to aid transparency. Participants should then be treated as multiple homogeneous panels and feedback generated and presented for each panel separately and criteria for retaining items based on individual panels. The development of the COS will be more robust and transparent if all stakeholders’ views are appropriately considered and valued at every stage, this should also improve subsequent uptake. If there are numerous panels, or some much smaller than others, one approach would be to define consensus with combination criteria. For example, an outcome must be scored 7–9 by at least 60 % of participants in at least three out of four stakeholder groups [35]. Further work is needed to determine the most appropriate way to do this.

## Discussion

This paper has explored the impact of analysing results from a Delphi survey by broad stakeholder groups (professionals and patients) and specific stakeholder subgroups, on the top 10 items prioritised for a COS. When the participants were considered as two broad stakeholder groups (two heterogeneous panels), two additional outcomes were prioritised compared to when participants were considered as a single heterogeneous group. Six further items were prioritised when participants were treated as multiple heterogeneous panels (professional and patient) (Table 3). Pooling broad stakeholder groups or all the participant data led to oversimplification of views of the study participants, with potentially important outcomes being lost. Decisions as to which stakeholders to include in a Delphi process and how to integrate them (in terms of single or multiple panels) in the presentation of feedback and consensus criteria are likely, therefore, to impact on the final COS. Indeed, donor site complications, an outcome that was only prioritised by one patient subgroup in the initial round of the Delphi process was ultimately included in the final COS for RBS [15].

Breast reconstruction is a particularly complex area, yet this exploratory work demonstrates the importance of a comprehensive sampling of stakeholders to include in a Delphi survey to select a COS. The application of qualitative techniques, such as maximum variation sampling with or without a sampling matrix, may provide a robust framework for this, although other methods may also be appropriate.

Other studies have demonstrated the importance of stakeholder selection in the development of COSs [9]. For example, when the set for rheumatoid arthritis was developed by the OMERACT group it did not include patients’ views [36–38]. Subsequent patient involvement demonstrated that fatigue was central from their perspective. This was added to the COS, highlighting, on a broad level, the importance of including all relevant stakeholder views [36]. Other Delphi surveys conducted as part of COS development have previously considered participants from professional and patient stakeholder groups and subgroups [14, 39, 40], but we are not aware of any that examined how this may influence selection of outcomes. Recent methodological work suggests that all participants should receive feedback for each stakeholder group [18] or stakeholder subgroup [14] separately to optimise consensus during the process of selecting COSs. This present study highlights differences in opinion across all of the stakeholder subgroups; presenting separate feedback for each subgroup enables participants to reconsider their scores in light of different views, hence enabling better consensus to follow. If stakeholder subgroups or broad groups are pooled, differences in opinion will be lost and potential consensus across stakeholder groups or subgroups reduced.

For the purposes of this methodological study we chose to rank outcomes based on the percentage of participants rating an item ‘extremely important’ (score of 9) and the top 10 outcomes identified. Core outcome set developers use a variety of consensus criteria within Delphi surveys for determining which outcomes should be included in the COS or taken forward to the next stage of the consensus process [17]. Commonly, these relate to a mean or median value for each outcome or a percentage of participants scoring an outcome as ‘important’. The top 10 was selected for this study to make comparisons across the different panels more straightforward; any other approach is likely to have identified similar differences in the outcomes prioritised.

Whilst this study suggests that all potentially relevant stakeholder subgroups should be considered as multiple homogeneous panels in a Delphi survey, it may not be possible to identify all the characteristics which influence outcome prioritisation. It may also not be feasible to adequately sample all identified stakeholder subgroups. Furthermore, different stakeholder characteristics (e.g. age and reconstruction type) may interact to influence which outcomes are valued. Such interactions may be difficult to disentangle in a Delphi process and preliminary qualitative work with stakeholders may be required to allow meaningful subgroups to be selected. In addition, it may be possible to conceive an almost infinite number of subgroups for a given condition. This would almost inevitably lead to under-representation of any single group and, depending on the criteria used to retain items, may lead to the retention of an unwieldy number of outcomes. Large numbers of subgroups may also complicate the provision of feedback in the Delphi process. Type of feedback has been shown to influence the prioritisation of outcomes in COS development [18]. How feedback is provided in the context of multiple stakeholder subgroups would, therefore, require careful consideration and further work is needed to determine how this process may be optimised. There is a need, therefore, to identify a priori the key professional and patient characteristics to consider as separate panels during the Delphi, ideally based on previous evidence. Breadth of experience can then be ensured with maximum variation sampling within each predefined subgroup.

It may also be argued that since the aim of the consensus methods is to determine a minimum ‘core’ outcome set, variation in outcome prioritisation by stakeholder subgroups at early stages in the process may be irrelevant since differences may disappear as the consensus process moves forward. However, if items are dropped early on because they are not deemed important by participants as a whole, or by all professionals or all patients; or if feedback is not presented for each subgroup separately, consensus across all stakeholders cannot be fully achieved.

Finally, this study may have implications beyond the Delphi process. One or more consensus meetings are an important step in agreeing and ratifying the final COS if uncertainty remains following the Delphi. Extrapolating the findings of this study, adequate representation from each identified stakeholder subgroup at these meeting would be vital to allow meaningful consensus to emerge. This may not be feasible if multiple stakeholder subgroups are involved, but should be advocated as best practice in future COS development studies when consensus meetings are considered necessary.

## Conclusions

Core outcome sets can improve the quality and consistency of research and, hence, its value to patients, professionals and policy-makers [10]. Careful and appropriate stakeholder selection and integration of stakeholder views, however, are necessary to ensure that the resultant COS is valid and accepted. Further work is required to produce definitive guidance on this important issue, but the formal, robust and transparent recommendations presented here may aid future COS developers in the design and conduct of their studies and may be the first step to improving the validity and value of COSs. Widespread adoption of these recommendations may, therefore, promote the uptake and use of future COSs in practice.

## Abbreviations

BRAVO: Breast reconstruction and valid outcomes
CNS: Clinical nurse specialists
COS: Core outcome set
RBS: Reconstructive breast surgery
